# Frontal EEG Temporal and Spectral Dynamics Similarity Analysis between Propofol and Desflurane Induced Anesthesia Using Hilbert-Huang Transform

**DOI:** 10.1155/2018/4939480

**Published:** 2018-07-15

**Authors:** Quan Liu, Li Ma, Shou-Zen Fan, Maysam F. Abbod, Qingsong Ai, Kun Chen, Jiann-Shing Shieh

**Affiliations:** ^1^School of Information Engineering, Wuhan University of Technology, Wuhan 430070, Hubei, China; ^2^Department of Anesthesiology, College of Medicine, National Taiwan University, Taipei 100, Taiwan; ^3^Department of Electronic and Computer Engineering, Brunel University London, Uxbridge, UB8 3PH, UK; ^4^Department of Mechanical Engineering, and Innovation Center for Big Data and Digital Convergence, Yuan Ze University, 135 Yuan-Tung Road, Chung-Li 32003, Taiwan

## Abstract

Electroencephalogram (EEG) signal analysis is commonly employed to extract information on the brain dynamics. It mainly targets brain status and communication, thus providing potential to trace differences in the brain's activity under different anesthetics. In this article, two kinds of gamma-amino butyric acid (type A -GABAA) dependent anesthetic agents, propofol and desflurane (28 and 23 patients), were studied and compared with respect to EEG spectrogram dynamics. Hilbert-Huang Transform (HHT) was employed to compute the time varying spectrum for different anesthetic levels in comparison with Fourier based method. Results show that the HHT method generates consistent band power (slow and alpha) dominance pattern as Fourier method does, but exhibits higher concentrated power distribution within each frequency band than the Fourier method during both drugs induced unconsciousness. HHT also finds slow and theta bands peak frequency with better convergence by standard deviation (propofol-slow: 0.46 to 0.24; theta: 1.42 to 0.79; desflurane-slow: 0.30 to 0.25; theta: 1.42 to 0.98) and a shift to relatively lower values for alpha band (propofol: 9.94 Hz to 10.33 Hz, desflurane 8.44 Hz to 8.84 Hz) than Fourier one. For different stage comparisons, although HHT shows significant alpha power increases during unconsciousness stage as the Fourier did previously, it finds no significant high frequency (low gamma) band power difference in propofol whereas it does in desflurane. In addition, when comparing the HHT results within two groups during unconsciousness, high beta band power in propofol is significantly larger than that of desflurane while delta band power behaves oppositely. In conclusion, this study convincingly shows that EEG analyzed here considerably differs between the HHT and Fourier method.

## 1. Introduction

Nowadays, it is widely accepted that anesthesia procedure plays an important role in everyday surgeries around the world. This kind of drug-induced unconsciousness can be reversible under anesthesiologists' manipulations [1]. Although anesthesia is essential for pain relief during surgery and other medical care procedures, it might potentially induce some adverse effects as well [2, 3]. Furthermore, anesthesia also could evoke some challenges such as emergence of delirium [4] and postoperative mortality [5, 6]. Clinically, professional anesthesiologists use vital signals (i.e., intermittent blood pressure, heart rate) to control the anesthetics titration procedure [7]. Studies on the complex brain dynamics under different anesthetic drugs might generally facilitate the theory of integrative processes that are very closely related to the anesthetic status [8]. In consequence, this would be used to optimize the anesthetics delivery in order to achieve ideal operation performance in regular anesthetic operations.

Propofol and desflurane belong to two kinds of commonly used anesthetics clinically through affecting the activity of inhibitory or excitatory cells to relieve pain [1, 9]. Propofol is the most commonly used anesthetic agent with rapid induction property. It mainly acts at gamma-amino butyric acid (type A, GABAA) receptors over the brain and spinal cord to increase the inhibition [10, 11]. It is known that GABAA neurons are widely located on the cortex, brainstem, thalamus, and spinal cord, and the changes induced by propofol can happen precisely and rapidly in arousal at multiple sites [12]. Propofol works for both sedation and maintenance of general anesthesia. In comparison with the intravenous propofol, desflurane belongs to GABAA agonist as well and known as commonly used inhaled anesthetics clinically. It exerts a more rapid onset of anesthesia features than other inhaled anesthetics like isoflurane [13]. Owing to hyperpolarization of the neurons, propofol induced unconsciousness is characterized in the electroencephalogram (EEG) by alpha (7.5-13 Hz) oscillations and coherence with delta (1-4 Hz) oscillations across the frontal cortex [14, 15].

It is regarded as evidence that propofol disrupts the connectivity between cortical regions. Similarly, it raises interests about the EEG dynamic changes under desflurane. Hence, comparing the EEG patterns between them may offer insights into investigating the neural circuits' mechanistic relationship [15]. EEG is measured from electrical potential in scalp. Because of anesthetics effects on the areas such as spinal cord, brain stem, or cortex [16, 17], EEG characteristics could provide meaningful information of brain rhythm activity [18]. It is widely acknowledged that central nervous system depends on neural spike or action potential to process the brain interaction [19]. It may be able to give some substantial explanations into the mechanisms of anesthetic action using EEG for analysis [2, 20].

EEG time frequency dynamics over the entire anesthesia procedure have been investigated in great depth. These studies are usually related to drug effect [21] and aging influence [2]. Many of them pursue the spatial distribution of the power changes, which comprehensively presents the brain-wide power fluctuations [22]. However, the key point is the selection of the various signal analysis methods. Most of the literatures are based on Fourier transform although some modified and improved versions are presented, e.g., multitaper method included in a previous study [23]. In that study, Fourier based spectrograms are presented to symbolize the temporal and spectral dynamics. However, since Fourier transform assumes the signal is stationary within very short window epochs, it can only be accurately applied to linear circumstances, whereas physiological signals are often nonstationary and nonlinear, which means previous results may not clearly exhibit the frequency band dominance patterns. Moreover, Fourier transform has a time and frequency uncertainty limit akin to the Heisenberg uncertainty theory in subject of quantum mechanics, i.e., tradeoff between time and frequency resolution, which means that it cannot generate high resolution results in both time and frequency domains simultaneously.

To be more explicit, a shorter window means higher time resolution but results in poorer frequency resolution. With longer window length, it will increase frequency resolution, but at the cost of time resolution [24]. Even the popular wavelet transform, essentially a kind of window adjustable Fourier transform, cannot avoid the limitation. Therefore, it cannot fulfill the strict requirement of EEG processing. In recent years, another empirically based data analysis method called Hilbert-Huang Transform (HHT) has received more and more attention [25]. It is realized by performing empirical mode decomposition (EMD) [26].

This idea is based on the iterative extraction of intrinsic mode function (IMF) and followed by Hilbert transform to calculate the spectrum. One of the most attractive advantages is that EMD could be applied to nonstationary and nonlinear data locally and adaptively without a priori knowledge about the system, overcoming the deficiency of pseudo-linearity and stationary signal assumption when Fourier transforms methods are used. It is conducted through sifting process to generate several IMF components from high frequency to low frequency. Thus, EMD based algorithms have been widely applied to noise reduction regardless of single or multichannel data [27, 28]. In addition, its instantaneous frequency is the derivative of the phase function and thus could define the local frequency rapidly. Furthermore, the Hilbert-Huang spectrum does not involve the concept of time and frequency resolution instead adopting the instantaneous frequency. Therefore, it is not limited to the Fourier time and frequency limit. Although EMD process is time consuming and it is sometimes difficult to determine the IMF combinations, it has been frequently applied to mechanical fault detection [29, 30], electromyography (EMG) analysis [31, 32] or EEG, and so forth. The details of the HHT method will be illustrated in methods section.

To explore the unknown brain circuit level mechanism related temporal and spectral properties more accurately, an intraoperative frontal electroencephalogram dynamics study using HHT method was performed under desflurane and propofol induced anesthesia separately. The time frequency patterns during different anesthetic stages are shown for comparisons. In order to make comparisons objectively and conveniently, the data for this study comes from our previous study [23], during which the traditional multitaper Fourier method is utilized. Comparison between two methods is undertaken as well to help discuss their results more reasonably.

## 2. Materials and Methods

### 2.1. Subjects and Anesthetic Procedure

This study was conducted in NTUH, where qualified patients were recruited to participate in this study. The Research Ethics Committee, National Taiwan University Hospital, Taiwan, assigned approval number 201302078RINC to this study. Patients were also required to sign the written informed consent in advance. Patients beyond 22 years old, without a neurological disorder or the history of obstructive sleep apnea and problems with anesthesia were selected for this project. Finally, 51 patients (propofol: 28 with 7 males; desflurane: 23 with 10 males) were eligible for EEG data collection. The demographics of the propofol group are shown as (mean ± std): age (yr): 50.7 ± 12.7, height (cm): 162.0 ± 9.4, weight (kg): 58.5 ± 11.0, and BMI (kg/m2): 22.2 ± 3.6. And desflurane group is with age (yr): 50.1 ± 15.0, height (cm): 160.1 ± 7.4, weight (kg): 57.7 ± 9.7, and BMI (kg/m2): 22.5 ± 3.1. Additional details are shown in [23]. Typically, the duration of operations was 76.0 ± 60.1 mins (propofol) and 138.7 ± 70.9 mins (desflurane), respectively.

Two group patients were not specifically selected, and they did not receive special treatment as well. All requirements were based on the routine operation criteria. Before operations, patients were required to keep limosis for at least 8 hours. Then, every patient received the appropriate volume of anesthetic agents to initiate the surgery. Subjects were confirmed to be fully anesthetized by both auditory stimuli and verbal response test during the study as well. Physiological anesthesia equipment was used to guarantee the surgery safety by monitoring EEG, electrocardiography (ECG), and photoplethysmography (PPG). Blood pressure, heart rate, and pulse rate were used as a reference for doctors' control as well. It can be categorized into 3 stages: awake, maintenance, and recovery. Intravenous propofol worked as induction agent due to its high effect speed. It was often combined with other adjunct analgesia drugs such as muscle relaxant medicine. Desflurane combined with air and oxygen was delivered by face mask for desflurane group whereas propofol was used for the propofol group during maintenance periods. Therefore, propofol worked along the whole surgeries in propofol group and desflurane was used to maintain the loss of consciousness (LoC) after the induction of propofol in desflurane group.

### 2.2. Data Recording and Preprocessing

**Figure 1:**
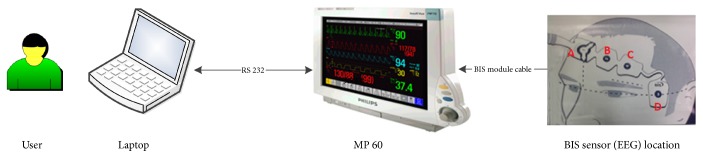
Experiment collection settings in the operation room. Adjunct sensors such as ECG and SPO2 are omitted intentionally. BIS sensor stripe must keep point A in the middle of forehead and point D horizontal with eye, and other points need to be flatly placed in the marked position in the picture.

MP60 anesthetic machine (Philips, Andover, MA, US) was used to monitor the patients' physiological signal. Factory default sensors were established before operation. For EEG recording step, special conductive paste was used to keep impedance under 5 k Ohm between skin and EEG sensor. EEG sensor is called BIS™ Quatro Sensor (Aspect Medical Systems, Newton, MA, US) and was connected to MP60 through BIS module, and then MP60 was connected with a laptop via a RS-232 serial communication cable, as shown in Figure 1. After the initialization of surgery, raw EEG signal and aforementioned signal were collected using a laptop. EEG and PPG were sampled at 128 Hz and it is 512 Hz for ECG. The intermittent signs such as BIS, blood pressure, heart rate, and SPO2 were recorded every 5 seconds. 2-min EEG data segments were visually chosen (free of artifact) to represent each stage. First, segments were selected under the eyes-closed awake state. Then, for LoC period of desflurane and propofol surgery, data segments were extracted in approximately middle part of surgery to guarantee the patients fully anesthetized, which also can eliminate the possible induction propofol effect on desflurane data at the same time. Once the patients begun response to doctor's voice stimuli, epochs would be determined as the recovery stage. After the preliminary selection, 4th-order Butterworth bandstop filter was applied to data, aimed at removing the 60 Hz line noise. Following the z-score normalization, the EMD worked as dyadic filter in the frequency domain [33]. The IMF1 was subtracted to remove the highest frequency components through the sifting process [34]. The remaining IMFs were used to conduct the time frequency analysis.

### 2.3. Hilbert-Huang Transform Analysis

The Hilbert-Huang Transform (HHT) was proposed by Huang et al. [35, 36]. It is the combination results of EMD and Hilbert spectral analysis (HSA) based on Hilbert transform (http://rcada.ncu.edu.tw/). IMF extraction procedure from signal is called sifting to achieve requirements: (1) the number of extrema and the number of zero-crossings must either be equal to or differ at most by one; (2) at any point, the mean value of the envelope both defined by the local maxima and minima should be zero. The procedure is shown below.

(1) Find all the local extrema and then generate the upper envelope by drawing a cubic spline line via all the local maxima; conduct the similar steps for the local minima to produce the lower envelope. The first component h_1_ is obtained by computing difference between the data and mean:

(1) $$\begin{array}{c} X\left(\;t\;\right)-m_{1}=h_{1} \end{array}$$

where *m*_1_ denotes the mean. h_1_ can only be treated as a proto-IMF. In the next step, h_1_ is treated as data:

(2) $$\begin{array}{c} h_{1}-m_{11}=h_{11} \end{array}$$

(2) After sifting up to k times, h_1_ becomes an IMF:

(3) $$\begin{array}{c} h_{1\left(k-1\right)}-m_{1k}=h_{1k} \end{array}$$

(3) Then, the 1st IMF is acquired as *h*_1*k*_:

(4) $$\begin{array}{c} C_{1}=h_{1k} \end{array}$$

(4) Repeat the steps above for residual generated by previous loop to get successive IMFs.

(5) Finally, signal is decomposed as

(5) $$\begin{array}{c} X\left(\;t\;\right)=\overset{N}{\underset{i=1}{\sum }}c_{i}\left(\;t\;\right)+r_{N}\left(\;t\;\right) \end{array}$$

where *N* is the number of IMFs, *c*_*i*_(*t*) is the ith IMF and *r*_*N*_(*t*) is the residual part. A combination of different IMFs can reconstruct a signal, thus eliminating the artifact.

As for nonlinear and nonstationary EEG signal analysis, unlike the traditional methods with constant amplitude and frequency in a harmonic component, an IMF represents a simple oscillatory mode as a counterpart and has varying amplitude and frequency over time. As a result, it applies the Hilbert transform in order to compute the instant frequency and amplitude, thus presenting more accurate spectral and temporal characteristics.

The Hilbert transform of *x*(*t*) can be treated as the convolution of *x*(*t*) with the function* h(t) *= 1/(*π*t). Since* t* convolution does not converge, the Hilbert transform is defined using the Cauchy principal value instead. Explicitly, the Hilbert transform of *x*(*t*) is defined as

(6) $$\begin{array}{c} \left(\;t\;\right)=\frac{P}{\pi }\overset{+\infty }{\underset{-\infty }{\int }}\frac{x\left(t\right)}{t-\tau }d\tau \end{array}$$

where* P* is the Cauchy Principal value.

Then, after coupling *x*(*t*) and *y*(*t*), the analytic signal *z*(*t*) can be obtained as

(7) $$\begin{array}{c} {z\left(t\right)=x\left(t\right)+i*y\left(t\right)=A\left(t\right)e}^{i\varphi \left(t\right)} \end{array}$$

(8) $$\begin{array}{c} A\left(\;t\;\right)=\sqrt{{x\left(t\right)}^{2}+{y\left(t\right)}^{2}}; \end{array}$$

where *A*(*t*) is the instantaneous amplitude of *x*(*t*), which can represent the signal's energy and *φ*(*t*) stands for the instantaneous phase of *x*(*t*).

Then, the time derivative of instantaneous phase *φ*(*t*) will be the physical meaning of instantaneous frequency *ω*(*t*) of signal *x*(*t*) in the following:

(9) $$\begin{array}{c} \omega \left(\;t\;\right)=\frac{d\varphi \left(t\right)}{dt} \end{array}$$

After applying the Hilbert transform to the EMD derived IMFs, HHT is obtained. Instantaneous frequency and amplitude in shape of a function of time can be shown in a contour map, designated as the Hilbert-Huang spectrum *H*(*ω*, *t*).

### 2.4. Statistical Analysis

**Figure 2:**
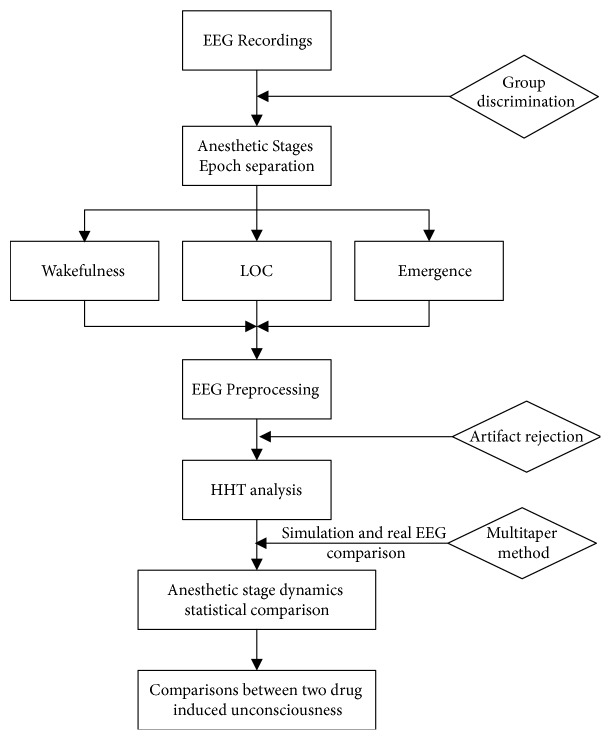
General analysis protocol in this study.

For each group comparison (i.e., dataset groups under propofol and desflurane or under different anesthetic states for individual drug where relevant), the group median outcome was taken among patients to present the time frequency variations. Also, each group median (IQR) power spectrum across frequency was computed from all involved patients' Hilbert edge spectrum. To evaluate the statistical significance for the difference in power at each frequency, a frequency domain-based bootstrapping algorithm was used to calculate the 95% (*α* = 0.05) confidence interval of the group median power spectra difference [37]. Fourier coefficients from normal Gaussian distribution with variance of its spectral power for each subject were calculated first, and then replicates of spectral power for each subject were computed from the Fourier coefficients and the median values across each group subjects were took to compute group difference. By repeating 2000 times, 95% confidence interval of group median power spectra difference was obtained at each frequency. The differences would be significant only if both the upper and lower bounds are present either above or below zero, which means the null hypothesis that the two group median power spectra are identical should be rejected [2]. Statistical analysis was undertaken by MATLAB (MathWorks, R2014b).  Figure 2 presents analysis protocol for the two groups.

## 3. Results

In this section, results of HHT on simulation data were presented first. By comparison with the traditional Fourier based Thomson's multitaper method implemented in Chronux toolbox (http://chronux.org) with parameters, window lengths of T = 2 s with 1.9 s overlap, time-bandwidth product TW = 2, and number of tapers K = 3. In addition, the difference on real EEG data under anesthesia is used to make comparison as well. Subsequently, we focused on HHT analysis related to specific pattern for different anesthetic stages under both propofol and desflurane, respectively. Comparisons within individual anesthetic agent over different stages are conducted. Moreover, the difference in LoC stages between the two specific drugs (propofol and desflurane) is shown.

### 3.1. Comparison between HHT and Multitaper Spectral Methods

#### 3.1.1. Simulated Synthesized Data

**Figure 3:**
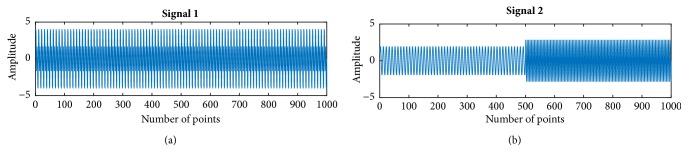
Two kinds of simulated signals. (a) Signal with fixed frequencies along entire signal lifetime; (b) signal with frequency shift over time.

**Figure 4:**
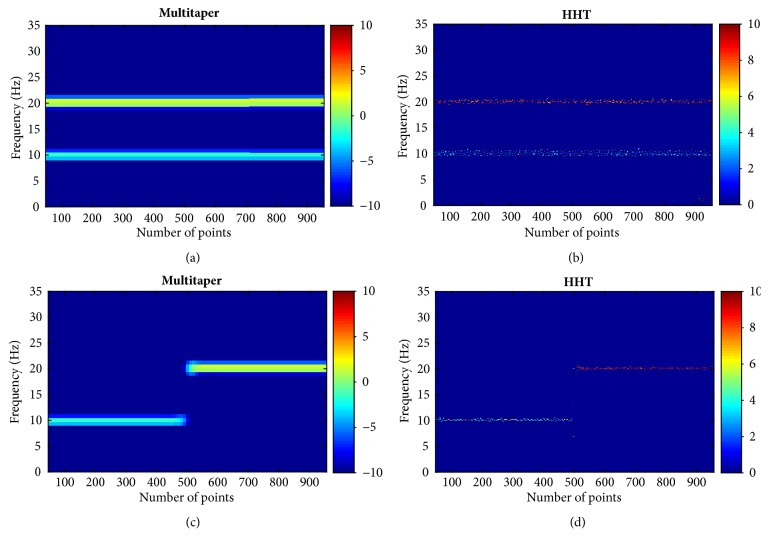
Time frequency plots of the two signals. (a) Traditional multitaper spectrogram of signal 1; (b) corresponding HHT of signal 1; (c) traditional multitaper spectrogram of signal 2; (d) corresponding HHT of signal 2. Note that the x-axis is number of points, which can be transformed into time values.

A simple simulated synthesized signal was created by composing two single frequency signals with different structure. Both consist of two sinusoidal components. One is the complex signal with two frequencies all over time (Figure 3(a)) and the other varies in frequency (Figure 3(b)). The sampling rate was set as 100 Hz. The two components frequencies were set as 20 Hz and 10 Hz, respectively. Then, HHT and multitaper spectrograms were applied to the simulated signal. Figures 4(a) and 4(b) give the multitaper and HHT spectrogram of signal 1. And the corresponding spectrograms of signal 2 are presented in Figures 4(c) and 4(d), respectively. Both methods can detect the real frequency of the combined signal. They exhibit the same characteristics of the spectrum. However, in this specific parameter determined example, the multitaper shows wider bandwidth for the high frequency components, which is similar to short time Fourier transform [34]. It can be also notably seen that HHT shows sharper spectrum dynamics and more resolved timing. Power of HHT does not keep constant across time. The ripple around the true frequency is consistent with the source signal amplitude fluctuation, whereas the Fourier assumes fixed sinusoidal components [30, 38, 39]. In the varied frequency simulation, Figures 4(c) and 4(d) show their spectrograms. The HHT can distinguish the frequency shift timing point without overlap. Similar to signal 1, even though signal 2 had some ripples, it may not result in serious statistical effect. Because it can be seen that the frequency with ripples oscillates around the real frequency value with narrower bandwidth, therefore, it is still thought that the estimated frequency can reflect the real frequency pattern of the analyzed signal in an average concept.

**Figure 5:**
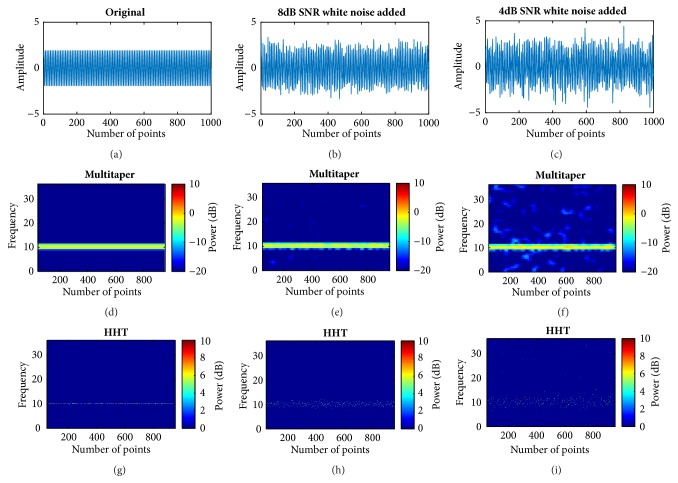
Time frequency plots of signals with white noise. (a) Original signal; (b) signal with 8dB SNR white noise added; (c) signal with 4dB SNR white noise added; (d) traditional multitaper spectrogram of original signal in (a); (e) traditional multitaper spectrogram of signal in (b); (f) traditional multitaper spectrogram of signal in (c); (g) corresponding HHT of original signal, (h) corresponding HHT of signal in (b); (i) corresponding HHT of signal in (c). SNR: signal to noise ratio.

In addition, simulations with 8 dB and 4 dB SNR white noise that were added to 10 Hz sine signal sampled at 100 Hz were undertaken. In Figure 5, the top row is signal waveform with increasing white noise from left to right (Figures 5(a), 5(b), and 5(c)) and middle and bottom rows are the corresponding multitaper and HHT results, respectively. It can be distinguished that HHT shows clear instant time frequency variations even with white noise. The edge of the original signal is much more distorted in multitaper result. Also, the average bandwidth of the combined signal is still sparse and narrow. Especially when comparing the white noise spectrogram outside the simulated frequency range, the bandwidth is much narrower in HHT.

#### 3.1.2. Propofol and Desflurane Induced Unconsciousness EEG Data

**Figure 6:**
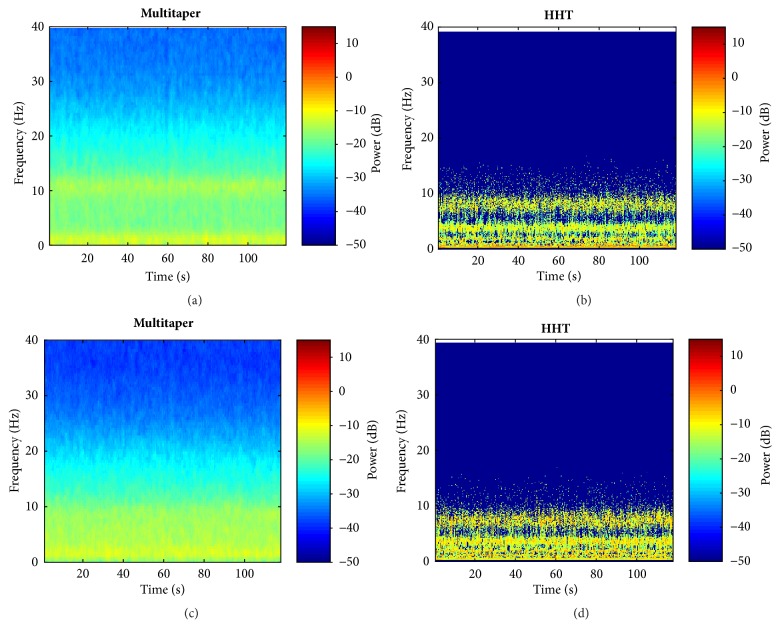
Comparison between the multitaper method and HHT using EEG data. (a, b) Traditional multitaper spectrogram and HHT of EEG under propofol maintenance, respectively; (c, d) traditional multitaper spectrogram and HHT of EEG under desflurane maintenance, respectively. Clearly, the HHT plots show sharper frequency energy distribution and clearer pattern.

In order to show the advantage of HHT over traditional Fourier based multitaper method in further, the anesthetized EEG data from propofol and desflurane group is used to compute the spectrogram. The multitaper and HHT results under propofol and desflurane are, respectively, shown in upper panels (Figures 6(a) and 6(b)) and lower panels (Figures 6(c) and 6(d)). Both methods exhibit similar pattern tendency for the two groups in general. It can be found that the slow and alpha band power was dominant. This makes sense in terms of consistency. Notably, desflurane group shows flatter band power distribution in multitaper plot than that of HHT, which makes it difficult to distinguish the band dominance pattern (Figures 6(c) versus 6(d)). In order to further clarify the better pattern distinction capability in HHT over multitaper, power variability (standard deviation) within each band that belongs to low frequency range (slow:<1 Hz, delta: 1 - 4 Hz, theta: 4 - 7.5 Hz, and alpha: 7.5 - 13 Hz) is calculated for both groups as shown in Table 1. From Table 1, results of multitaper are much smaller than that of HHT no matter under propofol or desflurane for all bands. It indicates the relatively uniform power distribution in multitaper, which makes it hard to distinguish the band power distribution feature under anesthesia. In contrast, HHT can be inferred that the power is converged to small bandwidth range, not evenly distributed across the entire specific band. This kind of better distinction performance of HHT is shown in Figure 6. Also, the band power peak frequency characteristic is investigated in Table 2. It can be seen that peak frequency distribution standard deviations of slow band and theta band in HHT indicate higher convergence in comparison with multitaper. Although little difference is found in delta band between the two methods, the alpha peak frequency generally decreases with minor standard deviation changes. In addition, for the two GABAA receptor dependent groups, HHT shows much more clear and similar consistency in time frequency dynamics patterns (Figures 6(b) and 6(d)), which may imply something in common between the two anesthetics. This is hard to find using the multitaper method (Figures 6(a) and 6(c)).

### 3.2. Propofol Group Temporal and Spectral Dynamics Comparisons across Brain States

**Figure 7:**
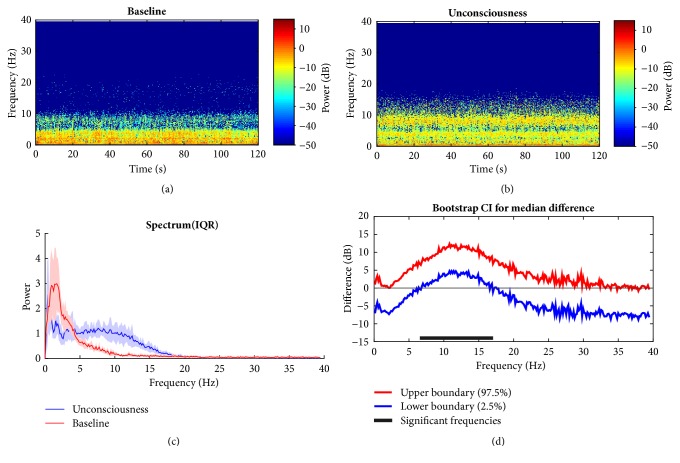
Comparison between baseline and anesthetized state under propofol. (a, b) Baseline and unconsciousness spectrogram; (c) median power spectrum distribution in group level. (d) 95% confidence interval of difference of median power spectra between two states with bootstrap analysis method. Solid line: median; shaded area: 25th–75th percentile IQR in (c). Horizontal solid black line: frequency range of significance. HHT: Hilbert-Huang Spectrum.

**Figure 8:**
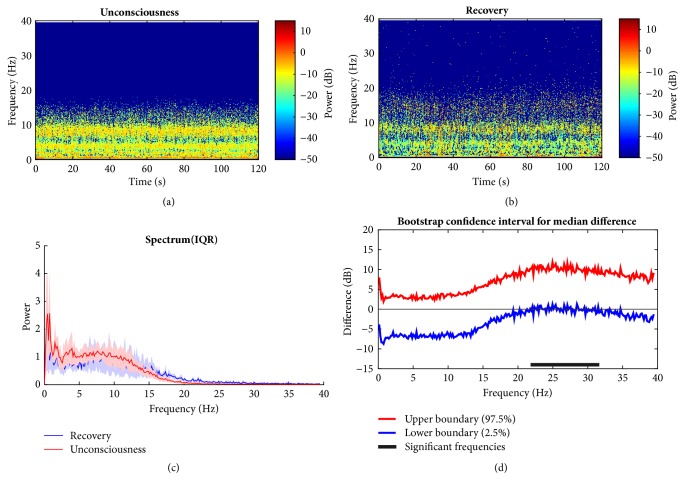
Comparison between recovery and anesthetized state under propofol. (a, b) Unconsciousness and recovery spectrograms; (c) median power spectrum. (d) 95% confidence interval of difference of median power spectra between two states with bootstrap analysis method. Generally, EEG power distribution is approximate between LoC and recovery, which may be due to the effect of propofol. In beta band, it shows significant power increase, which indicates the high oscillation function when awake.

To find the signature of EEG in propofol group, the HHT method described above is employed by taking its nonstationary properties advantage into consideration. The evident differences in the time frequency distribution are observed. Figures 7(a) and 7(b) show the outcome of baseline (awake state) and the unconsciousness (anesthetized state), respectively. During the baseline, the dominant power is located in the frequency bands (i.e., slow wave, delta band) below 4 Hz (also seen in Figure 7(c)). For the unconscious period, it is clear that alpha band emerges and the slow wave and delta band power relatively decline compared with the wakefulness. This is consistent with findings in [21, 40]. Power in delta and alpha bands is dominant during propofol anesthesia. High frequency band (beta) power remains approximately unchanged between two states (Figure 7(c)). From statistic test, the null hypothesis that the group median power spectra across specific frequency band are the same is rejected if the confidence interval bounds are either both above or below zero; i.e., the band median power is significantly different between two groups. Hence, alpha and low beta band power (Figure 7(d): 6.8 Hz to 16.7 Hz) are significantly larger during LoC. As to the comparison between the recovery and anesthetized state, Figure 8 is presented to illustrate the variation. It is just a little different between unconscious state and recovery. During recovery period, an approximately similar pattern appears like that of unconscious state with relatively lower energy across different bands. This may be owing to the anesthetic effect duration. However, it can be preliminarily found that the high rhythm returns, which occurs during human's conscious state. This means when recovering, the null hypothesis is rejected for low gamma band comparison, which implies the significant power increase whereas other bands do not (Figure 8(d)).

### 3.3. Desflurane Group Temporal and Spectral Dynamics Comparisons across Brain States

**Figure 9:**
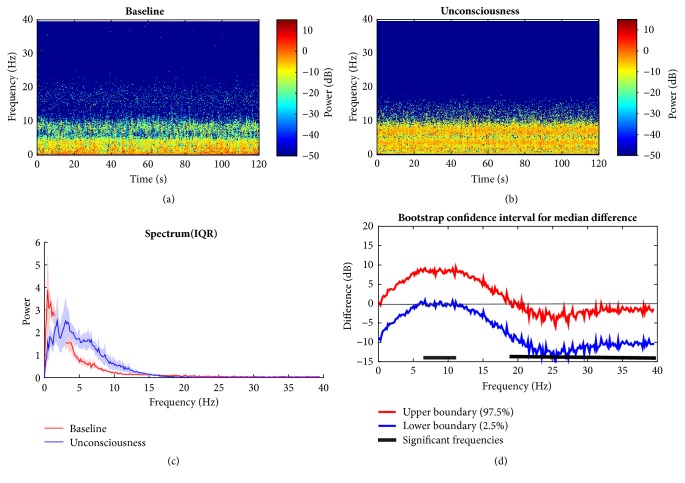
Comparison between baseline and unconsciousness under desflurane. (a, b) Awake baseline and unconsciousness spectrogram; (c) median power spectrum. (d) 95% confidence interval of difference with paired bootstrap analysis method between two states median power spectra. Significance is found for increased alpha band power and decreased beta and gamma band power during loss of consciousness.

**Figure 10:**
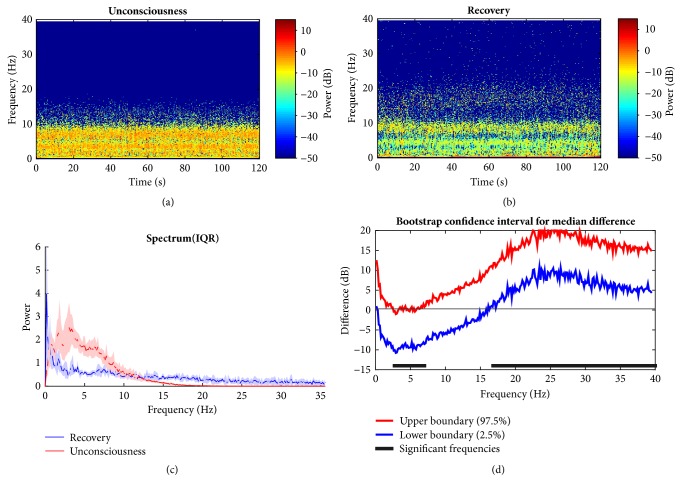
Comparison between unconsciousness and recovery for desflurane group. (a, b) Unconsciousness and recovery spectrograms; (c) median power spectrum. (d) 95% confidence interval of difference of median power spectra between two states with bootstrap analysis method. The high beta and gamma ranges show LoC state power is significantly higher during recovery state, while other low bands keep relatively weaker within theta band. Overall, the recovery state resembles that in awake state in Figure 9.

Desflurane, another kind of commonly used GABAA dependent anesthetic agent, is studied across different stages. By contrast, the median spectrogram during unconsciousness keeps delta and alpha band power dominant, both of which are greatly enhanced (Figures 9(a) and 9(b)). The power spectra IQR distribution can verify this pattern (Figure 9(c)). However, the slow wave decreases a lot under anesthesia in comparison with baseline. Bootstrap analysis about group median power spectra difference statistics over frequency presents significant higher alpha (6.7 Hz to 10.9 Hz) while delta does not, and high beta and gamma power decrease significantly during unconsciousness 19.02 Hz to 40 Hz (Figure 9(d)). As for the comparison between anesthetized state and recovery, they behave in similar pattern with regard to band power distribution with overall lower strength during recovery (Figure 10). This may be in part due to residual anesthetic effect. They exhibit similar band power distribution generally. The low frequency power within theta decreases significantly during recovery (Figure 10(d): 3 Hz to 7 Hz). However, the high beta and gamma band power reverse with significantly growing strength from 16.8 Hz to 40 Hz (Figure 10(d)), which is consistent with comparison within the propofol group in Figure 9. It means this band power grows higher, which is intended to return baseline.

### 3.4. Spectral Analysis Comparisons between Propofol and Desflurane under Anesthesia

**Figure 11:**
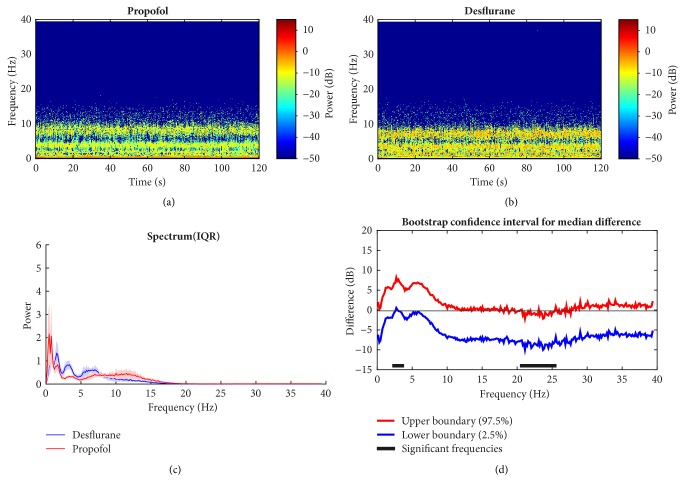
Temporal and spectral dynamics between propofol and desflurane group during unconsciousness. (a, b) Propofol and desflurane group, respectively; (c) median power spectrum; (d) 95% confidence interval of difference of median power spectra between two groups with bootstrap analysis method. Significant difference within high beta band is observed.

Since the two agents share the same molecular principle, the temporal and spectral dynamics under anesthesia are of great interest to explore. Through observations, rough similarities are found between them. They both show clear alpha and delta band power dominance (Figures 11(a) and 11(b)). Both groups are characterized with three peak bands, which are slow wave, delta, and alpha bands (Figure 11(c)). It may be due to some consistent anesthetic effects on brain oscillations and neural interactions. However, it can be shown that there is higher spectral power in desflurane across low frequency. A short band power within delta range in desflurane group is significantly higher than that of propofol (Figure 11(d): 2.3 Hz to 3.5). This can also be observed in Figure 11(c). On the contrary, its high beta band power of propofol is significantly higher than that in desflurane using bootstrap statistical analysis rejecting the null hypothesis which both group median power spectra are identical across frequency. In short, the basic patterns of both these common GABAA anesthetics behave similarly even though some statistical differences occur in delta and high beta bands.

## 4. Discussion

This study mainly focuses on single frontal channel EEG spectrum related analysis under the two commonly used anesthetics induced unconsciousness (propofol and desflurane). The outcome provides some insights into specific EEG signatures, which correlate with specific drugs using nonstationary method HHT.

The most widespread approach to assess the anesthetic state depth is based on commercial indices with a single number between 0 and 100 generated from the EEG [3, 41, 42]. Some other researchers use approximate entropy derived analysis to characterize the anesthetic effect [43]. However, it is hard to make a universal conclusion to represent anesthetic states with only such a single number. Since anesthesia is a complicated pharmacological phenomenon, these indices are influenced by multiple factors, including neurologic disorders such as dementia [44], hypothermia [45], or noise evoked by muscle, eye blink, and electrical perturbation. Raw EEG related time frequency analysis spectrogram has been proposed to reflect the anesthetic status [12]. It would be interesting to investigate the whole frequency band of interest variation patterns over time. Different oscillation behaviors could provide potentials to study the cerebral mechanism under anesthesia.

Some prior works have focused on analysis of single specific anesthetic agent [12, 46]. It is worthwhile using advanced algorithms to study the comparison between these two drugs although some research groups have made spectrogram relevant comparisons between propofol and desflurane [47–49]. It is noticed that both propofol and desflurane generally appear similar in the spectrogram fluctuations. They exhibit strong alpha and slow band power dominance during loss of consciousness, and the alpha band results are strongly consistent with previous literature [23]. There should be some shared function principles between the two drugs. However, except for alpha band presence, it is quite interesting that the desflurane shows relatively higher delta band power than alpha band power. Their relative power proportion is much closer than that in the propofol group, which presents the difference of drug effect on the brain activities.

Nevertheless, propofol and desflurane are GABAA receptor dependent. As we know, some NMDA types such as ketamine and nitrous oxide function with different mechanism [46]. Even for the commercially single index monitors, they are still far from assessing anesthetic status precisely for both GABAA and NMDA categories. However, HHT-derived spectrogram might be applied to investigate the specific EEG related signatures among them [12, 46, 50]. It may provide potentials to look into their unique characteristics. Besides, connectivity analysis such as coherent oscillations and phase lag also has attracted much attention to study the correlation between frequency bands recently [15, 21, 51]. These methodologies can provide new thoughts on interpretation about the differences between both awake and unconscious states if utilized appropriately.

Furthermore, this advanced spectrogram generation method is based on the EMD and Hilbert transform as described in the introduction. The advantages have been clearly presented in simulation signals. It has no traditional concept of frequency resolution, which is especially appropriate to nonstationary data. Therefore, the results could precisely present the features related to frequency band power distributions. Table 1 shows the narrower bands in HHT than FFT, as known that HHT is a two-step data analysis technique (EMD and Hilbert transform). It utilizes the concept of instantaneous frequency and the derivative of phase. It decomposes signal into different IMF without a priori conditions, unlike FFT which is based on linear concept and stationary assumption after choosing basic functions. FFT is represented by the combinations of many basic sinusoidal functions and its harmonics whereas the HHT does not. Therefore, harmonics show more evident impacts on FFT other than HHT. FFT spectral energy at the base frequency is easily distributed to the higher frequency component and HHT energy remains in the neighborhood of the base frequency [52]. Thus, Hilbert transform allows for the concentration of frequency energy and the avoidance of the uncertainty principle [53]. In Figure 6 and Table 2, it is found that the peak alpha frequency differs with lower value by HHT. From perspective of clinics, Brown's group pointed out that brain oscillations generally change from low-amplitude, high frequency bands to high-amplitude, low frequency ones at LoC [1, 12]. This phenomenon particularly happens with GABAA dependent anesthetic agents. Similar brain oscillation fluctuation patterns were found by Mashour [54]. This somehow in turn explains the clear occurrence of theta band power in HHT though traditional method does not. However, it may be also due to the HHT method principal. Because HHT uses sifting procedure to decompose the signal into different IMFs, it may result in artifactual introduction, thus generating spectral peaks shifts of the main location of frequency bands. It should be explored further in terms of the mathematical principal. Another interesting point is that the HHT results found under the two anesthetics induced unconsciousness are much more similar (Figures 6(b) and 6(d)). This may be due to some GABAA drug pharmacological action principle, which propofol and desflurane both belong to. This provides a potential to observe similarities about the brain's activity under GABAA receptors dependent drugs induced anesthesia whereas it is not clearly shown in multitaper method. In comparison to other literature [39], HHT shows evident theta band changes. For the desflurane cohort, HHT method clearly demonstrates its similar pattern to that in propofol, which proves its superior capability to be applied to the analysis over multiple anesthetic agents. However, in our previous paper [23], the desflurane pattern has not been clearly shown regarding the low frequency part using the traditional method. This perhaps gives more exploration potentials within the low frequency component for specific drugs.

As to the stage comparisons in each group, there is a significant power increase in alpha power from awake state to unconsciousness. However, the high beta and gamma band significance is found only in the desflurane group instead of propofol whereas this significance is found in both drug groups in the previous multitaper work [23]. The significance finding in multitaper is partly due to cleanness of the data segment selection visually without high frequency noise removal, which is done in HHT procedure and reduces the EEG difference within high beta and gamma bands between awake and unconscious states. As to the difference in two groups, it may be due to the different properties of two drugs. As it is known, propofol clinically produces sedation most of the time, which suppresses the high frequency components less than desflurane does. In other words, desflurane can produce stronger neuro communication disruption than propofol. This may be inferred that the high beta and gamma bands brain activities are much more suppressed, thus resulting in the significant difference within high frequency band in desflurane instead of propofol. This is also found in Figure 11, where high beta band power of propofol is significantly higher than that of desflurane. Another possible reason is that HHT mathematically changes the main frequency shift when decomposing the signal into IMFs during sifting procedure, which is seen in the comparison of Figure 6. This probably changes the band power distribution to some degree.

Some limitations may still exist in this study. First, our analysis focuses on the single frontal channel data due to the easy access in clinical surgery settings. However, it is much worthwhile to investigate spatial variability [12, 15, 55]. For example, anteriorization of alpha power is observed in propofol cohort [22, 55], and the cross-channel connectivity is attracting more attention to anesthesia related brain network during anesthesia. High-density EEG investigations using methods such as transfer entropy and granger causality are employed to detect the spatial correlation [56, 57], which further helps exploring the neural circuit mechanisms within these oscillations patterns. Secondly, our data amount is relatively small and specifications are not strictly required due to our data acquisition conditions in regular surgery settings, during which combination of analgesia and muscle relaxants must be coadministrated. It also means all the surgeries are scheduled for general patients receiving operations, not particularly designed for the anesthesia study analysis; regular standard of procedure was undertaken as usual. As a result, it was not possible to obtain exactly proper concentration-EEG response curve. All actions by the doctors were done to keep the surgeries run smoothly without any consideration of our study. However, as inspired by ideas proposed by Akeju et al. and Pal et al. under strict drug conditions [46, 58], their works are regarded with great value. Based on our data properties, it is interesting to investigate whether these consistent findings are available in regular surgeries that need general anesthesia. It is of great importance to verify this in practical standard of procedure during surgeries. Thus, HHT and Fourier methods are implemented to study this issue. Meanwhile, this has explored the differences between the two methods. Results are positive and worthwhile to continue.

In future, records such as agent concentration levels, anesthetic levels should be specified. Moreover, several drawbacks of HHT have been studied such as edge effects and intensive computation time consumption. Researchers proposed that independent component analysis (ICA) combined with EMD could effectively eliminate the edge effect [32, 59] and local mean decomposition (LMD) is proved to provide better performance than EMD when combined with ICA [60]. Moreover, EMD uses sifting process to generate IMFs until they meet the decomposition requirements; however, this may lead to artifactual introduction such as border effect. It may also lead to frequency shifts of the main location of frequency bands, which may explain reason of the band peak frequency movement compared to previous work [23] (e.g., lower peak frequency shift in alpha). This needs to be validated in future. Finally, it is ideal to clarify the whole operation dynamics over time. The present results are obtained through segment. Although intentional visual inspection and EMD filtering were applied, it is obviously not sufficient and precise to remove the noise. Beyond the traditional Kalman filter, new advanced filtering methods have come into existence. For example, ICA based algorithms are recently popularly applied to biomedical signal [61–63], which efficiently removes the eye blink or muscle noise in real time. Therefore, new advanced noise removal methods need to be explored to facilitate our analysis protocol.

## 5. Conclusions

In this study, compared to traditional multitaper Fourier transform method based spectrogram patterns during unconscious state, HHT method exhibits spectrogram distribution with higher band power convergence and shows theta band presence pattern. Frontal EEG exhibits increased alpha band power occurrence in both propofol and desflurane induced unconsciousness when compared to awake state using HHT method. It provides potential for anesthesiologists to observe EEG spectrogram signatures in real-time mode, thus interpreting the anesthetic specifications. HHT-derived spectrograms seem to be a promising approach to gain insights concerning the common brain neural circuit mechanisms underlying these two typical GABAA receptors dependent drug-induced unconsciousness states. In addition, the slight differences between the HHT spectrograms associated with these two anesthetic agents may unfold a better understanding of the neurophysiological mechanisms that distinguish them. In the future, it might prove beneficial to integrate neurophysiological principles and brain network connectivity together, targeting the construction of an anesthetic-dosing dependent model.

## Figures and Tables

**Table 1:** Frequency band power distribution variation. Standard deviation (std) of power within each frequency band is calculated for every case among both methods and then all the standard deviation values in a specific group are statistically shown as mean ± std.

		**Slow (< 1 Hz)**	**Delta (1 - 4 Hz)**	**Theta (4 - 7.5 Hz)**	**Alpha (7.5 - 13 Hz)**
**Propofol**	Multitaper	0.011 ± 0.007	0.015 ± 0.008	0.002 ± 0.001	0.009 ± 0.005
	HHT	1.130 ± 1.063	0.235 ± 0.106	0.089 ± 0.026	0.206 ± 0.612

**Desflurane**	Multitaper	0.013 ± 0.010	0.014 ± 0.011	0.007 ± 0.006	0.010 ± 0.005
	HHT	0.922 ± 1.883	0.352 ± 0.239	0.225 ± 0.265	0.071 ± 0.031

**Table 2:** Peak frequency distribution characteristics across different bands. Values shown as mean ± std (Hz).

		**Slow (< 1 Hz)**	**Delta (1 - 4 Hz)**	**Theta (4 - 7.5 Hz)**	**Alpha (7.5 - 13 Hz)**
**Propofol**	Multitaper	0.30 ± 0.46	1.48 ± 0.10	6.12 ± 1.42	10.33 ± 1.16
	HHT	0.61 ± 0.24	1.46 ± 0.13	7.15 ± 0.79	9.94 ± 1.17

**Desflurane**	Multitaper	0.88 ± 0.30	1.66 ± 0.56	6.01 ± 1.42	8.84 ± 0.82
	HHT	0.74 ± 0.25	1.61 ± 0.44	6.33 ± 0.98	8.44 ± 0.74

## Data Availability

The data used to support the findings of this study are available from the corresponding author upon request.
